# Human-centric predictive model of task difficulty for human-in-the-loop control tasks

**DOI:** 10.1371/journal.pone.0195053

**Published:** 2018-04-05

**Authors:** Ziheng Wang, Ann Majewicz Fey

**Affiliations:** 1 Department of Mechanical Engineering, The University of Texas at Dallas, Richardson, TX 75080, United States of America; 2 Department of Surgery, UT Southwestern Medical Center, Dallas, TX 75390, United States of America; University of Vermont, UNITED STATES

## Abstract

Quantitatively measuring the difficulty of a manipulation task in human-in-the-loop control systems is ill-defined. Currently, systems are typically evaluated through task-specific performance measures and post-experiment user surveys; however, these methods do not capture the real-time experience of human users. In this study, we propose to analyze and predict the difficulty of a bivariate pointing task, with a haptic device interface, using human-centric measurement data in terms of cognition, physical effort, and motion kinematics. Noninvasive sensors were used to record the multimodal response of human user for 14 subjects performing the task. A data-driven approach for predicting task difficulty was implemented based on several task-independent metrics. We compare four possible models for predicting task difficulty to evaluated the roles of the various types of metrics, including: (I) a movement time model, (II) a fusion model using both physiological and kinematic metrics, (III) a model only with kinematic metrics, and (IV) a model only with physiological metrics. The results show significant correlation between task difficulty and the user sensorimotor response. The fusion model, integrating user physiology and motion kinematics, provided the best estimate of task difficulty (*R*^2^ = 0.927), followed by a model using only kinematic metrics (*R*^2^ = 0.921). Both models were better predictors of task difficulty than the movement time model (*R*^2^ = 0.847), derived from Fitt’s law, a well studied difficulty model for human psychomotor control.

## Introduction

Human-in-the-loop robot-assisted systems have substantial freedom in design that enable operators to interact with complex physical systems in a variety of ways. In the case of teleoperated robotic systems, users can control robot end-effectors through position-based teleoperation [1], or use asymmetric teleoperation methods, where the master and slave systems have different degrees of mobility (i.e. user inputs, system inputs, and system outputs have different degrees of freedom) [2]. A classic example of an asymmetric teleoperation control scheme is the control of multiple agents (e.g., unmanned aerial vehicles) [3, 4], with a single user input. Another example is controlling nonhonolonomic systems such as wheelchairs [5] or steerable needles [6] through desired end-effector positions in Cartesian space, rather than through joint space control of system inputs (i.e. velocity and steering angle). Finally, techniques are also now available to allow both human and robot some level of autonomy while sharing control of the overall task [7, 8].

With all this flexibility in the design of human-in-the-loop control systems, an important research question arises: how should one design these interfaces to be intuitive and easy to use, and how can the assessment of this control effort be quantified for complex tasks?

Substantial efforts have been made to quantify, or model, difficulty in general human-machine interaction. The most well-known theory among these is Fitts’ law [9], a predictive model of human motor systems. Fitts’ law is a widely accepted theory which can describe human psychomotor behavior in a simple bi-variate pointing task. In a Fitts’ task, participants are required to move as fast as possible between two targets of a certain width, W, separated by a distance, D. The task index of difficulty, ID, can be mathematically quantified by a log-linear relationship between the distance, D, and the target width, W. The units of this index of difficulty are called “bits”. This phenomenon is formalized as *MT* = *a* + *b* × *ID*, where *MT* is the movement time, and difficulty levels are quantified as the index of difficulty (*ID*), which can be mathematically determined by: *ID* = log_2_(2*D*/*W*).

Fitts’ difficulty model has been primarily used in the evaluation of human-computer interfaces and for ergonomic applications [10–12]. The Fitts’ law model has also been shown to apply to complex tool manipulation tasks, such as those required in surgery. A work by Lin, *et al.* explored the validity of Fitts’ law in addressing laparoscopic instrument manipulation while performing laparoscopic surgery [13]. Chien, *et al.* investigated the relationship of speed and accuracy in robot-assisted surgery, and suggested its roles in association with surgical skills [14].

However, conventional Fitts’ law may not be sufficient to evaluate the difficulty of more complex human-in-the-loop control tasks, particularly in teleoperated or shared-control scenarios where the user’s task objectives are not known to the robotic system. Fitts’ law simply quantifies the relationship between the difficulty of the task and the movement time required to finish it. Recently, a few studies have begun to reveal correlations between the difficulty index of a task and changes in human user motion dynamics, in both discrete and cyclical movements [15, 16]. Still, there is an opportunity to further explore how changes in task difficulty affect the user response, globally, including cognitive, physiological, and kinematic changes. These insights could lead to the improved design of human-in-the-loop control algorithms for complex tasks, such as the teleoperation of robotic systems.

As a step towards this goal, this paper presents an data-driven approach to assess the difficulty of typical reaching task, based on objective, task-independent measures of human cognition, physical effort, and motion characteristics. We hypothesize that Fitts’ law will be preserved in robot-assisted control interfaces, meaning that we expect an increase of movement time using a haptic device with increasing difficulty levels. Furthermore, we propose that the difficulty of a reaching task can be objectively quantified from multiple measures of user sensorimotor response, including user physiology and motion kinematic metrics found in both the user (limb motions) and task (tool motions) workspaces.

This paper is organized as follows: First, we provide a review on current difficulty assessment tools and existing techniques for human response recognition. Second, we describe our experimental protocol, including the experimental task design, signal acquisition and sensor benchmarking. Next, we introduces methods used to extract important modeling features and technique used for modeling. Further, we present statistical analysis results of proposed features, and the performance of our models to predict task difficulty. Last, detailed explantation of results and discussions on the limitations, potential use, and future work are presented. Finally, we conclude our study.

## Background and related work

### Current methods to assess task difficulty

In general, the difficulty of a human-in-the-loop control task, such as teleoperation, is difficult to define. It is a common practice to evaluate teleoperation difficulty and performance via quantitative tool-based metrics and performance measures, such as completion time and manipulation accuracy [17, 18]. Also, these metrics are often coupled with checklists or user-response surveys, such as NASA Task Load Index (NASA-TLX) [19], where operators report the ease-of-use and acceptance of the system by rating it on predefined scales. These ratings have been widely used to assess task workload and perceived performance in a variety of domains, including robotic surgery [20–23], and teleoperation [24].

However, these evaluations are limited because: (1) task-specific performance measures do not always correspond to perceived user acceptance and performance [6, 25–28]; (2) user ratings can only be measured in a post-hoc manner, and do not capture the real-time response of the human user.

### Objective human response recognition

Over the years, several techniques have been proposed for objectively measuring a human users physiological response in terms of affective state, motivation, environment awareness, and mental and physical workload, to name a few [29–34]. These studies typically leverage the use of sensors such as electroencephalography (EEG), surface electromyography (EMG), galvanic skin response (GSR), and heart response (HR). Electroencephalography (EEG) is an objective measure of neurophysiological changes related to electrical activity of brain neocortex. This technique enables researchers to quantitatively study human emotion, perception, cognition and technical skills [22, 35–37]. Surface electromyography (EMG) measures signals of electrical activity that are generated by active muscles. Analysis of EMG response is substantially helpful in revealing underlying motor patterns, physical effort, and user motion intent prediction [38, 39]. Galvanic skin response (GSR), also known as skin conductance response, is a quantitative measure of electrical conductance fluctuations on the skin surface during a period of time. It is regarded as a reliable indicator of the individual’s cognition load, attention, and emotional states [40]. Skin conductance increases due to an increase of moisture on the skin when the individual is under stress, and vice versa. Measurements of heart rate and its variability (HRV) include the electrocardiograms (EKG) or photoplethysmography (PPG). Heart activity has been shown to capture the dynamic workload, emotion, and cumulative stress [41].

In addition to physiological response, metrics derived from human movement sensors have also shown to be able to capture important information regarding the ability of the human user to perform a motor task [42, 43]. Orientation-based motion metrics were able to discriminate expert from novice surgeons in robotic and open needle driving [44]. Estrada, *et al.* developed smoothness assessment on tool motion data, and reported that motion kinematics in endovascular tasks showed significant correlations with participants’ surgical skills [45]. Kinematic profiles of user movement also demonstrated as an objective tool to assess technical skills and performance in surgical tasks [46, 47]. Nisky, *et al.* explored the effects of teleoperation and expertise on the kinematics of user joint movement [48–50].

## Experimental methods

An IRB approval (UTD IRB #14-57) was obtained through the University of Texas at Dallas IRB office. In this paper, we aim to develop predictive models of task difficulty that do not depend on task-based metrics, such as movement time and targeting error. Rather, metrics derived from the user physiological response and kinematic movements could prove to be better predictors of how difficult the task is.

### Experiment protocol

A bivariate target-reaching task was developed in a simulated virtual environment. Participants were instructed to perform the reaching task by using their dominant hand to manipulate a virtual tool to reach predefined target locations. To control the position and orientation of the tool in the virtual reality, a 6 degree-of-freedom haptic device, the Phantom Omni (Geomagic Touch, 3D Systems, SC, USA), was used. This device provides 3-degree-of-freedom force feedback and 6 degree-of-freedom sensing. A custom C++ code was developed to randomly generate targets within the virtual experiment, rendered by the CHAI3D haptic library. To constrain movement in 2D task workspace, a virtual haptic wall was created, where the haptic gain (k) was set as *k* = 150.

**Fig 1:**
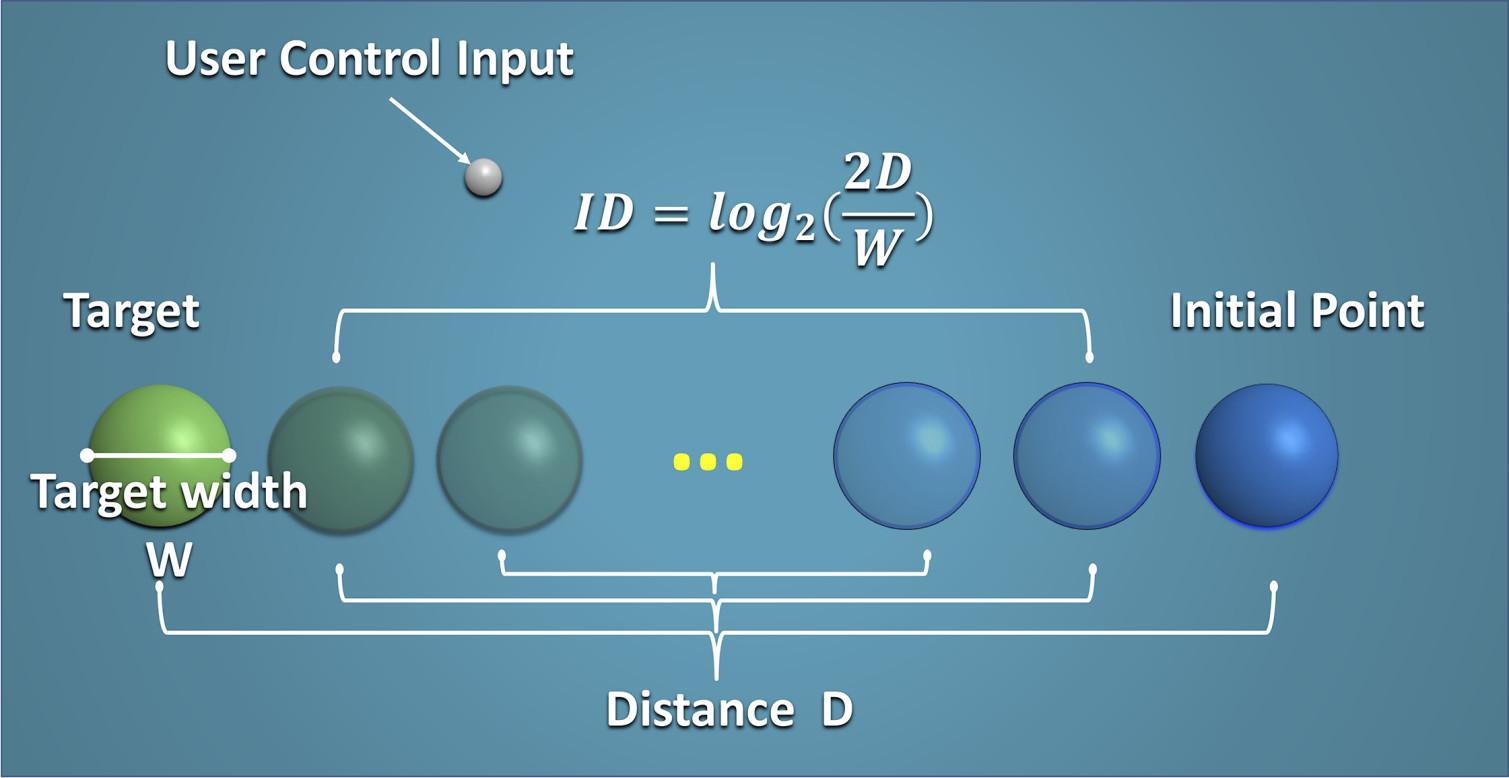
Simulated target-reaching in virtual reality with user control input. Tasks with known indices of difficulty (IDs) were associated with varied distances (D), as defined by Fitts’ law.

In order to create different difficulty conditions in the experiment, the distances between the starting and final target were changed, according to Fitts’ law (Fig 1). The target width, W, was set to 5mm, and the target distances varied, including: 10mm, 20mm, 40mm, 80mm, 180mm, and 320mm. The combination of the target width and different distances resulted in reaching tasks of 6 different difficulty conditions, with the index of difficulty (ID) ranging from 2.0 to 7.0 bits.

The experiment consisted of a training session with 6 unrecorded exercises and a formal testing session. In the training session, subjects practiced the reaching task in our simulation and learned how to move the stylus and advance the experiment. To initialize each task, participants were asked to hold the stylus in the center of the workspace, resulting in a neutral position for the shoulder: a 90° elbow flexion, 90° forearm pronation, and a neutral wrist. Then, they moved to the starting target. Data started recording when users hit the initial starting point. After training exercises, participants performed the reaching tasks in five randomized, blocked repetitions for six different conditions of difficulty, resulting in a total of 30 trials for each subject. In order to avoid biasing due to the potential learning effects, all task conditions and target locations were randomly chosen. In each block, participants were asked to reach each of the six targets in 5 repeated cycles. This was done to ensure sufficient data collection time for the targets with the lowest index of difficulty. The subjects were explicitly asked to manipulate the haptic device freely and in a natural way, and informed that the experiment had no time or performance requirements.

### Participants

A total of fourteen subjects (11 males and 3 female: mean age = 21 years, *SD* = ±6 years) participated in this study (recruitment date: January 10, 2017). All subjects provided informed written consent in accordance with The University of Texas at Dallas Institutional Review Board (UTD IRB #14-57). The individual in this manuscript has given written informed consent (as outlined in PLOS consent form) to publish these case details. Participants had no previously reported muscular-skeletal injuries or diseases, or neurological disorders.

### Sensor data acquisition

**Fig 2:**
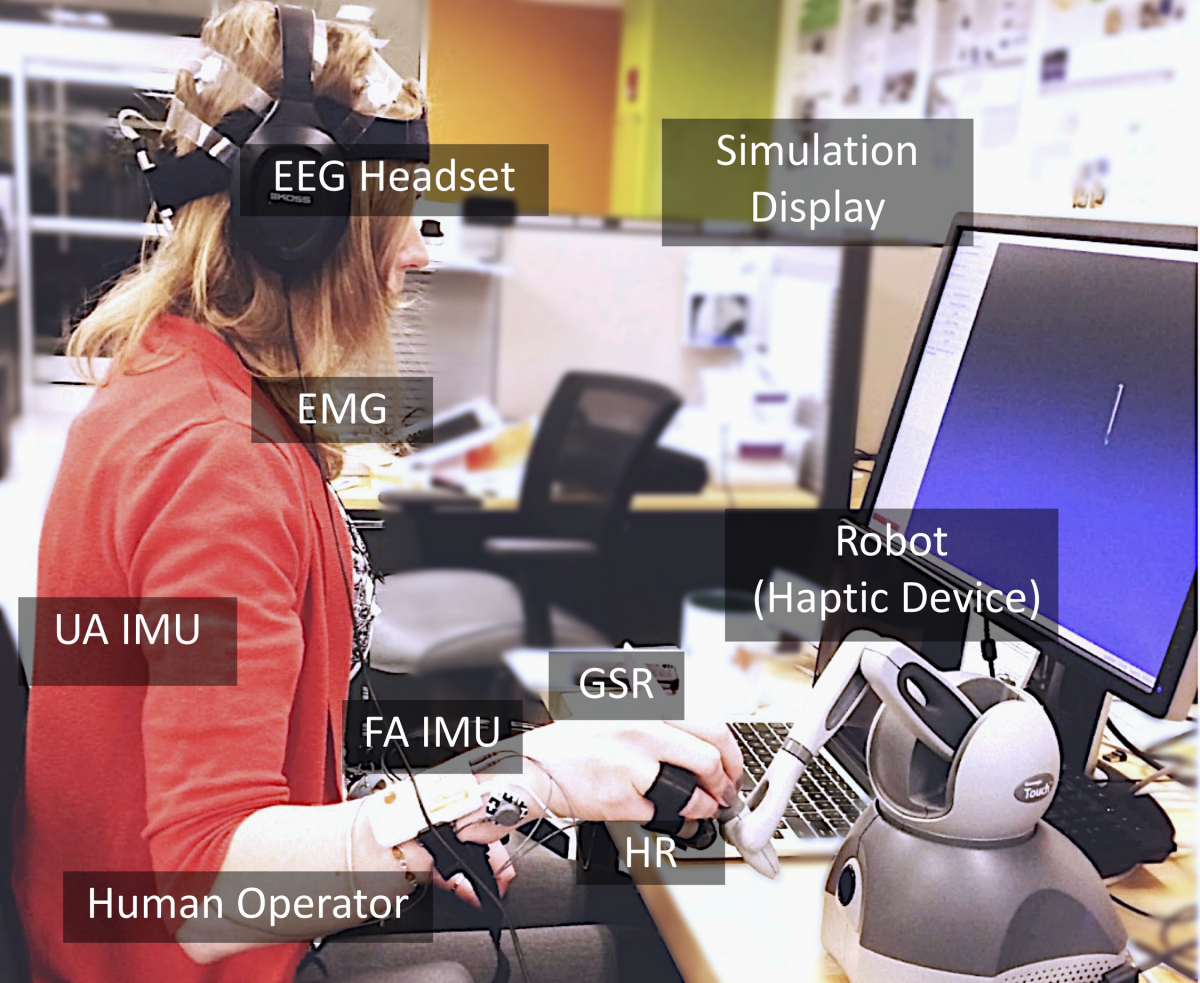
Instrumentation for experiments. Subjects interacted with the virtual task by controlling a robotic interface (i.e., haptic device). Physiological response (e.g., EEG, GSR, EMG) and user kinematic movements were recorded from wireless inertial measurement units (IMUs) on the upper and forearm, as well as encoder readings from the haptic device.

**Fig 3:**
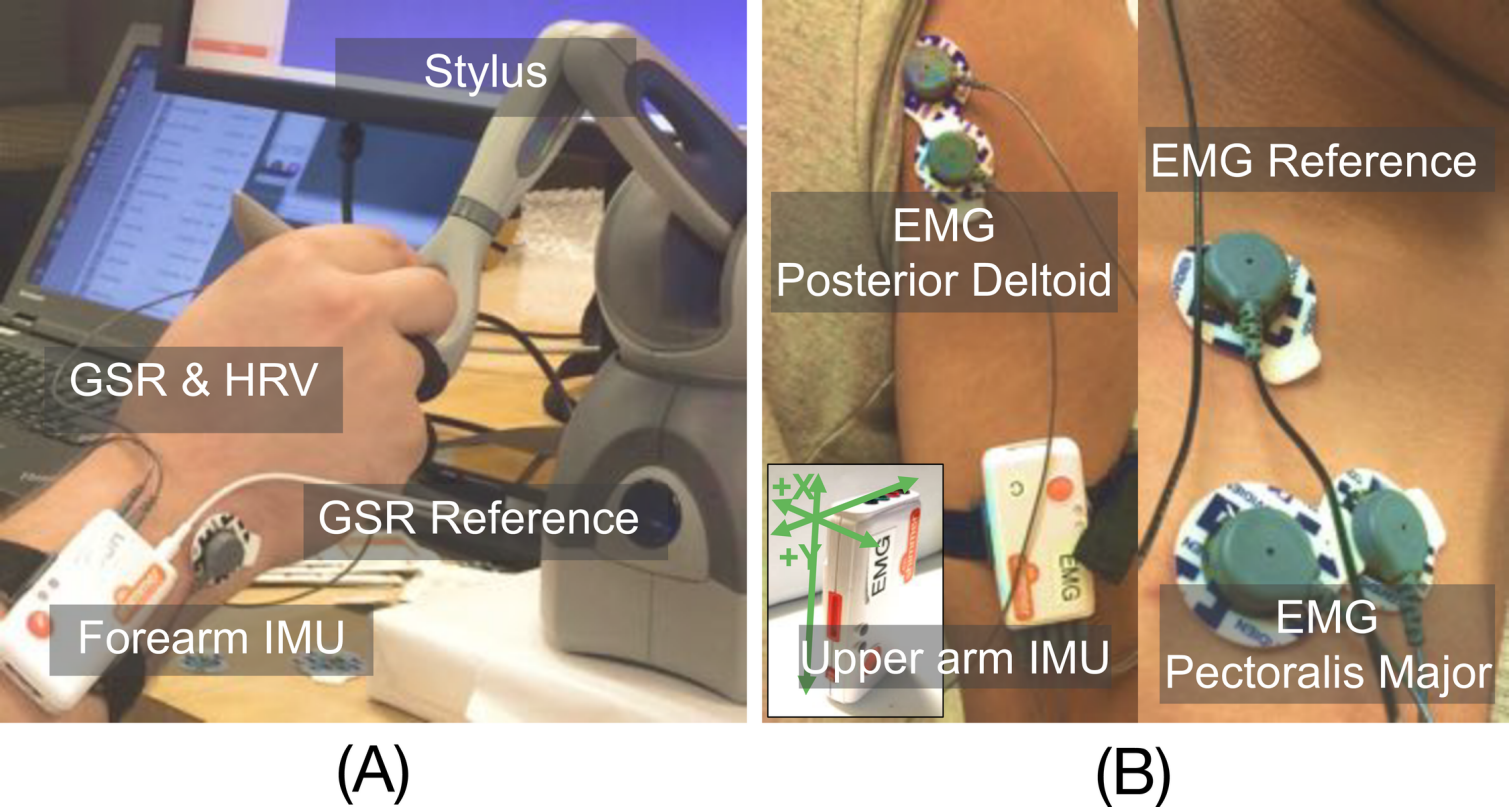
Sensor placement. (A) a sensor attached on the user forearm measuring forearm (FA) IMU, GSR and fingertip PPG signals; (B) a sensor attached on the upper arm measuring upper-arm (UA) IMU and 2-channel EMG signals.

To capture human response during the manipulation task, a custom multi-channel sensor data acquisition system was developed. Fig 2 shows a subject interacted with the acquisition system via controlling a robotic interface. Real-time physiological response and user motor kinematics were recorded using the Robot Operating System (ROS) framework. Additionally, to minimize external distractions, noise-canceling headphones playing white noise were used. The sensor configuration and placement is shown in Fig 3.

To monitor muscle activity, surface EMG signals were collected using Shimmer Sensing toolkit at the sampling rate of 1024 Hz, and streamed wirelessly via Bluetooth. Two muscles, Pectoralis Major (PectM) and Deltoid Posterior (PostD), were selected for EMG data collection. These muscles are primarily active during internal and external rotations of the shoulder (internal rotation rounds the shoulder in, while external rotation rounds the shoulder back), and were found to be most active during the bivariate pointing task. Two Al/AgCl electrodes (2 cm apart) were placed on the chest wall, 2 cm below the collarbone to capture PectM muscular activity; another one pair of electrodes are placed 2 cm below the lateral border of the scapular spine and parallel to muscular fibers to obtain PostD muscular signals [51]. As EMG signals can vary across individuals, we acquired the maximum voluntary isometric contraction (MVIC) level for each muscle. Each subject was asked to contract muscle as strongly as possible and to hold the contraction for 30 seconds. To test the pectoralis major, subjects stood straight facing the wall with elbow bent at 90-degree angle and pressed their hand against the wall. Next, subjects were asked to rotate their arm and pressed the back of hand against the wall to get the deltoid posterior MVIC. This procedure was repeated three times, with two-minute intervals between MVIC trials to relax the muscle. The highest peak value of the three iterations was chosen as a reference MVIC EMG value for each muscle.

EEG signals were collected using the BIOPAC^®^ B-Alert X10 wireless EEG system with AcqKnowledge^®^ software. The sensor headset includes 10 Al/AgCl scalp electrodes, and data was sampled at 1000 Hz. The 10 sensors were placed in locations on the mid-line and lateral EEG sites, *F*3, *Fz*, *F*4, *C*3, *Cz*, *C*4, *P*3, *POz*, *P*4, as recommended by the sensor manufacturer [52]. Furthermore, the EEG signals were benchmarked to obtain the baseline data of BIOPAC EEG bio-metrics for the individual subject. The EEG benchmark test consisted of three sessions (15 minutes in duration): a three-choice psychomotor vigilance task (3C-VT), a visual psychomotor vigilance task (VPVT), and an auditory psychomotor vigilance task (APVT) [53, 54].

The heart rate of each subject was acquired using fingertip photoplethysmography (PPG) [55, 56] (a.k.a., optical pulse measurement), included with one of the Simmer Sensing units. The optical pulse sensor was attached to the ring finger tip of the dominant hand. For monitoring user GSR signals, the Shimmer sensor was also used to measure the skin conductance between the index and middle fingers. Both the GSR and PPG signals were sampled at a frequency of 512 Hz, and transmitted by a Shimmer Sensing sensor to the data acquisition computer. To normalize the GSR and heart response among each subject, a benchmarking session was performed wherein subjects were asked to close their eyes and stay relaxed while listening to white noise for 3-minute duration. The baseline heart-rate and variance in skin conductance was recorded from this session.

## Methodology

### Feature processing and extraction

A variety of metrics were generated to describe motor difficulty with respect to the human physiological response and movement during each trial. These metrics are independent of the type of task, and are inspired from literature, as described in the following subsections.

#### Physiological response metrics

Physiological response metrics were generated to describe the user experience in terms of cognition, attention, and physical effort. These metrics include those derived from EMG muscle activity, EEG cognitive state, skin conductance and heart response signals.

The raw EMG signals were digitally filtered (4th-order FIR filter) with a bandpass frequency from 20 Hz to 450 Hz to attenuate motion artifacts. A 60 Hz notch filter was also applied to remove unwanted power line interference. In addition, the DC component of EMG signal was removed by subtracting the average of the signal. Two time-domain features of the EMG signals were extracted: the mean absolute value (MAV) and the root-mean-square, both were normalized using the MVIC reference value for each subject.

Mean absolute value of EMG is suggested to be an optimal detector of EMG amplitude, commonly used in EMG pattern-recognition and myoelectic control [57, 58]. As an extension of the regular mean-absolute value calculation, the second-type MAV has a smoother weighted function, allowing for improved accuracy. This type of MAV is defined as an average of weighted rectified EMG signal amplitude. The mathematical calculation of MAV is expressed as:

$$\begin{array}{ccc} MAV & = & \frac{1}{N}\sum_{i=1}^{N}w_{i}\left|x_{i}\right|\\ w_{i} & = & \left\{ \begin{array}{cc} 1, & \text{if}\;0.25N\leq i\leq 0.75N\\ \frac{4i}{N}, & \text{elseif}\;i<0.25N\\ \frac{4(i-N)}{N}, & \text{otherwise} \end{array}\right. \end{array}$$ (1)

where |*x*_*i*_| is *i*-th sample of the rectified EMG signal segment, *N* is the signal length, and *w*_*i*_ is the piecewise weighted function.

Root-mean-square (RMS) is another commonly used feature in EMG signal analysis, useful to reveal force patterns and muscle contraction activation [57, 59]. The RMS of EMG was calculated using a 2ms moving averaging window based on the rectified envelope of EMG signals. The mathematical definition of RMS is expressed as:

$$\begin{array}{c} RMS=\sqrt{\frac{1}{N}\sum_{i=1}^{N}|x_{i}{|}^{2}} \end{array}$$ (2)

where |*x*_*i*_| is *i*-th segment of rectified EMG signals with length of *N*.

For the EEG measurements, five cognitive parameters were obtained from BIOPAC Cognitive State Analysis software. During each reaching trial, the average probabilities of cognition states are computed through continuous wireless EEG recording at 1 Hz. The cognitive states include: Engagement, Workload, Distraction, SleepOnset, Head Movement Level (HeadMvL). These metrics are classified from EEG raw signals, and normalized as probability measures compared to the EEG benchmarking assessment of each individual subject. Potential contamination from movement artifacts and eye blinking are identified and avoided via filtering methods [60]. Details regarding this EEG signal processing technique and validation of the cognitive state metrics can be found in the literature [53].

The sampled GSR signal was filtered using a 2nd-order lowpass filter with the cut-off frequency at 5 Hz to reduce unwanted muscle artifacts (high frequency noise). To avoid potential phase distortion, the GSR signal was preprocessed with a FIR filter. The variance of the galvanic skin conductance, *SC*_*vr*_, defined as the difference between the global maxima and minima of GSR signals during each task trial, was computed and normalized using the baseline variance measurement for each subject. [61].

Heart rate response was acquired using photoplethysmography (PPG) of user’s fingertip. The raw PPG signals were filtered by a FIR filter to remove noise and obtain a linear envelope of PPG signal. The heart rate was calculated based on the peak-to-peak intervals (PPI) of PPG linear envelopes. The calculated heart rate was normalized for each subject by the average heart rate measured in the baseline task.

#### Kinematic motion analysis

In order to assess the ease of the haptic device manipulation task, kinematic motion metrics were derived in both the task (i.e., haptic device) and user (i.e., limb) workspaces.

**Fig 4:**
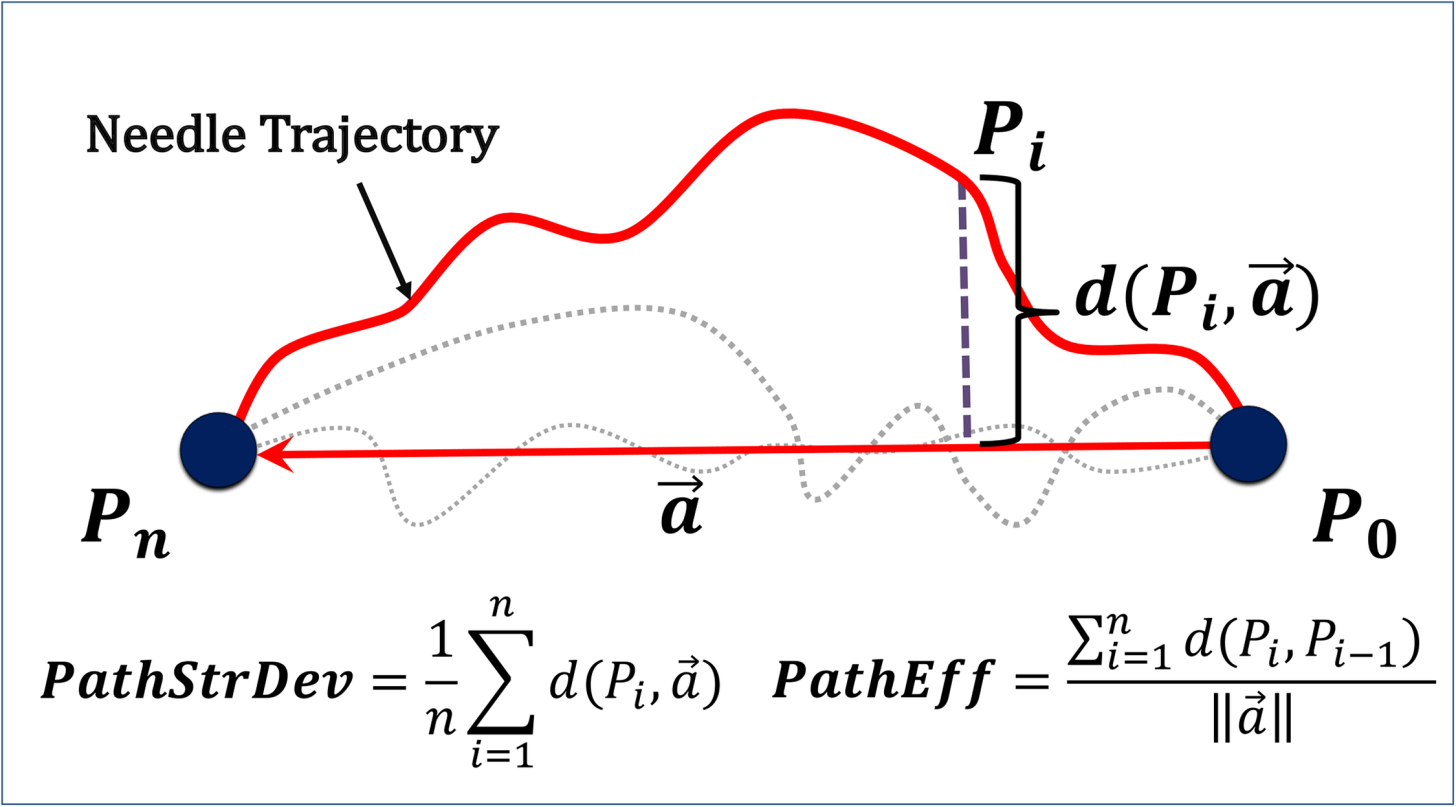
Path straight deviation (*PathStrDev*) and path efficiency (*PathEff*) metrics. These measures are obtained from end-effector trajectories in the task space.

In the simulated task space, we extracted two task-independent metrics from position measurements of the end-effector. These metrics include path straight deviation, *PathStrDev*, and path efficiency, *PathEff*, as shown in Fig 4. Path straight deviation (*PathStrDev*) is defined as the average magnitude of the orthogonal projection of the current position, *p*_*i*_, onto the vector $\vec{a}$ between the initial user position, *p*_0_, and the end user position, *p*_*n*_. The mathematical calculation of *PathStrDev* is expressed as:

$$\begin{array}{c} PathStrDev=\frac{1}{n}\sum_{i=1}^{n}d(P_{i},\vec{a})=\frac{1}{n}\sum_{i=1}^{n}\frac{∥\vec{P_{0}P_{i}}\times {\vec{a}}^{\;}∥}{∥{\vec{a}}^{\;}∥} \end{array}$$ (3)

The path straight deviation quantifies the trajectory straightness compared to the purely straight path between the starting and final positions. This metric is task-independent since it is computed based on the user-defined end-point, not the target end-point. The path efficiency (*PathEff*) is calculated via dividing the total path-length of the tool by the straight line distance between the initial and end position of the user. *PathEff* has been reported as a measure of the user’s ability to continuously control the end-effector [62, 63].

To characterize limb motion in the user space, we generated three types of motion metric using the data from inertial measurement units (IMUs) on both forearm (FA) and upper arm (UA). These metric including: the average magnitude of the angular velocity (*AngVel*), linear acceleration (*LinAcc*), and the root-mean-square magnitude of jerk (*Jerk*). The resultant jerk, *J*, as a function of the time derivative of acceleration, is defined by:

$$\begin{array}{c} J=\sqrt{{\left(\frac{d^{3}x\left(t\right)}{dt^{3}}\right)}^{2}+{\left(\frac{d^{3}y\left(t\right)}{dt^{3}}\right)}^{2}+{\left(\frac{d^{3}z\left(t\right)}{dt^{3}}\right)}^{2}} \end{array}$$ (4)

where *x*, *y*, *z* are the three-dimensional components of the trajectory in Cartesian space. The effects of gravity on the linear acceleration measurements obtained from the IMUs were eliminated by calculating the acceleration magnitudes for each time-sampled *x*, *y*, *z* components. In this study, the root-mean-squared magnitudes of jerk were computed in order to reduce the variability and noise of measurements, by averaging over the entire waveforms. Based on the literature, the jerk metric is a valid candidate for assessing movement smoothness, which can be interpreted as a general measure of overall control ability [64, 65].

### Data analysis and statistics

A Pearson product-moment correlation analysis was performed to assess the correlation between the index of difficulty and extracted explanatory features. Correlation coefficients were calculated using the data across all subjects and repetitions. A value of correlation coefficient *r* above 0.60 was considered to be a strong correlation, a value between 0.30 and 0.59 was considered a moderate correlation, and an *r* value between 0.20 and 0.29 was considered a weak correlation. In addition, a one-way analysis of variance (ANOVA) was conducted to test for correlation between task difficulty and the proposed dependent variables. A significant correlation was determined by a *p*-value less than 0.05.

### Modeling task difficulty

Metrics extracted in the previous section were used to quantify reaching tasks with different indices of difficulty. Four candidate models were generated, using a regression technique, to evaluate the ability of kinematic and physiological response metrics to predict task difficulty, when compared to movement time. This type of modeling is important as movement time in teleoperated and shared control systems is ill-defined (i.e., the robotic system has no knowledge of when the user ends a given task).

#### Feature selection and metric sets for modeling

To determine the best subsets of metrics, a feature selection process was carried out using the Pearson correlation criteria. Candidate metrics with no correlation or very weak correlation to task difficulty were excluded to reduce dataset uncertainty. Metrics with correlation coefficients below 0.20 were removed from further modeling.

Four sets of metrics were chosen for each of the four models generated. Specifically, we were interested in evaluating the ability of both physiological and kinematic metrics to predict task difficulty when compared to movement time, which is the only metric used in the Fitts’ law model. For each model, a different set of metrics was used including only movement time (Set *I*), all metrics with weak correlations to task difficulty or greater (Set *II*), only kinematic metrics (Set *III*), and only physiological response metrics (Set *IV*).

#### Metric normalization

Due to the differences in dynamic ranges and various units of input metrics, the raw data was pre-processed by a normalization process using a *z*-score transformation. For a sampled variable *x*_*i*_ with mean *μ* and standard deviation *σ* over *n* instances, the *z*-score for each data point is calculated as:

$$\begin{array}{c} z_{i}=\frac{x_{i}-\mu }{\sigma } \end{array}$$ (5)

where *x*_*i*_ is the *i*-th data point of *n* sample instances.

#### Partial least square regression

A multivariate data-based approach, Partial Least Square Regression (PLS-R), was chosen to generate each of the four models. The general aim of partial least square regression model is to explain the information of the task difficulty index (responses) by using the multiple characteristics of human response (predictor variables) as input. The underlying calculation of PLS-R is formulated as:

$$\begin{array}{ccc} X & = & TP^{T}+E\\ Y & = & UQ^{T}+F \end{array}$$ (6)

where ***Y*** is the matrix of responses, ***X*** is the matrix of input predictor variables; ***T*** and ***U*** are the scores, or latent matrices by decomposing or projecting ***X*** and ***Y***, respectively; ***P*** and ***Q*** are orthogonal factor matrices; ***E*** and ***F*** are the error residuals matrices, which are assumed to be random normally distributed. Mathematically, the regression of partial least square was achieved by maximizing the covariance structures between the two scores matrices, ***T*** and ***U***, so as to maximize the covariance between responses (***Y***-matrix, ID) and all possible linear combination of predictor variables (***X***-matrix).

In general, PLS regression can produce more reliable models compared to other standard regression methods, such as multiple linear regression. PLS methods are particularly suitable dealing with high-dimensional and noisy data, handling a larger number of predictor variables with a small set of observations. Additionally, PLS regression allows a multivariate modeling, while dealing with the potential problem of multicollinearity, which is often the case in multivariate datasets.

#### Training, testing and evaluation

For the purpose of a valid prediction and non-biased assessment without overfitting, a *k*-fold (*k* = 10) cross-validation (CV) technique was employed. In the *k*-fold cross validation, normalized data samples were randomly partitioned into *k* non-overlapping subsets with equal sample sizes. The holdout process of cross-validation was repeated *k*-times. Of the *k*−th partitions, one single subset of observations were used for testing, while the union of remaining *k* − 1 subsets would form a set for training. The CV estimates of overall accuracy were acquired by averaging of all the *k*-fold individual measures to obtain a reliable assessment.

To assess the predictive performance, models in both training and testing steps were evaluated based on the following deterministic criteria: the Coefficient of Determination (*R*^2^), Mean Absolute Percentage Error (*MAPE*), Root Mean Squared Error (*RMSE*), Mean Absolute Error (*MAV*). In addition to these scale-dependent accuracy measures, non-unit accuracy metrics, Normalized Root Mean Squared Error (*NRMSE*), Normalized Mean Absolute Error (*NMAV*), were obtained for accessing non-unit magnitudes of residual errors, which gave a idea of the relative differences between the modeled and observed value. Mathematical definition and descriptions of deterministic criteria are given in Table 1.

**Table 1:** The deterministic criteria used to assess model predictive performance.

Criteria	Mathematics	Description
***R*^2^**	$\frac{\sum_{i=1}^{n}{\left(y_{i}-{\bar{y}}_{i}\right)}^{2}-\sum_{i=1}^{n}{\left(y_{i}-{\hat{y}}_{i}\right)}^{2}}{\sum_{i=1}^{n}{\left(y_{i}-{\bar{y}}_{i}\right)}^{2}}$	Coefficient of determination, *R*^2^, quantifies the goodness-of-fit. The value of *R*^2^ ranges from to 1, higher value indicates a better model fit.
**RMSE**	$\sqrt{\sum_{i=1}^{n}{\left(y_{i}-\hat{y_{i}}\right)}^{2}}$	*Scale-dependent prediction accuracy measure. Root mean squared error between predicted and observed response values, in the same unit of ID.*
**MAE**	$\frac{1}{n}\sum_{i=1}^{n}|y_{i}-\hat{y_{i}}|$	*Scale-dependent prediction accuracy measure. MAE measures average absolute errors between predicted and observed response values, in the same unit of ID.*
**NRMSE**	$\frac{1}{y_{max}}\sqrt{\frac{1}{n}\sum_{i=1}^{n}{\left(y_{i}-\hat{y_{i}}\right)}^{2}}$	*Normalized non-unit root mean squared error between the predicted and observed response values. The value of NRMSE closing to zero suggests higher prediction accuracy.*
**NMAE**	$\frac{1}{n}\sum_{i=1}^{n}\frac{|y_{i}-\hat{y_{i}}|}{y_{max}}$	*Normalized non-unit average of absolute error, measure of prediction accuracy independent of scale.*
**MAPE**	$\frac{1}{n}\sum_{i=1}^{n}\frac{|y_{i}-\hat{y_{i}}|}{y_{i}}*100\%$	*Mean absolute percentage error, MAPE, measure of prediction accuracy as percentage.*

The *y*_*i*_, $\hat{y_{i}}$ are observed and estimated response values, ${\bar{y}}_{i}$, *y*_*max*_ are the mean and maximum of the observed response, and *n* is the size of sample instances.

## Results

### Statistical analysis

**Fig 5:**
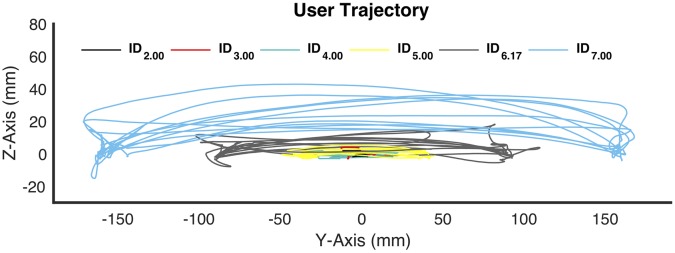
All end-effector trajectories in task space controlled by a typical subject. Different colors denote target-reaching tasks with different indices of difficulty.

**Fig 6:**
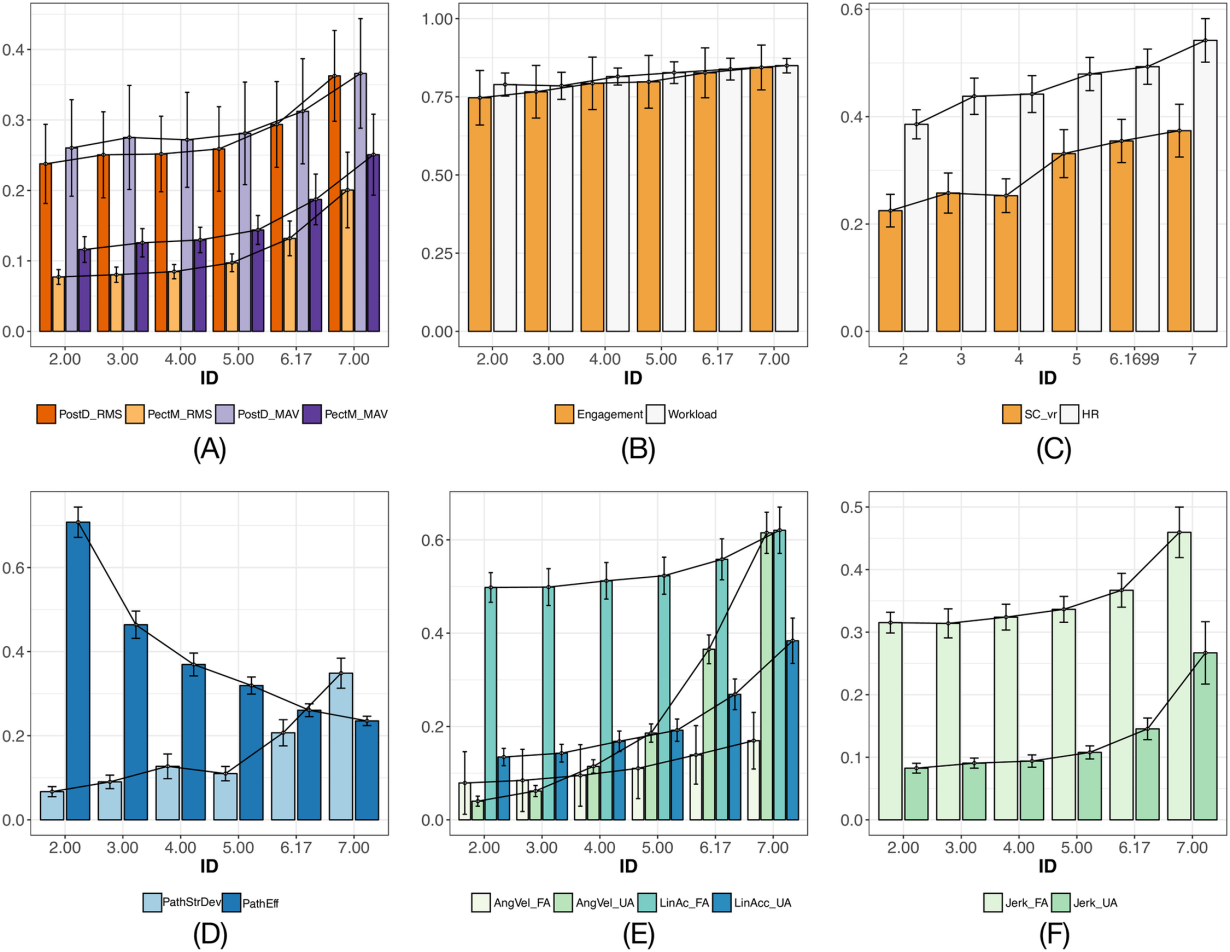
Overall results of human response for all reaching tasks with different difficulty indices (IDs). (A) EMG muscle activity, (B) EEG-based cognition, (C) galvanic skin conductance variance and heart rate response, (D) movement of end-effector in task space, (E) and (F) user limb motion on both forearm (FA) and upper arm (UA). Black lines are connecting mean values of corresponding measures, error bars illustrate the variances in a 95% confidence interval.

Example user trajectories, obtained from the position data of haptic device end-effector, are shown in Fig 5. Fig 6 shows all of the measured outcome variables, extracted from EMG, EEG, GSR, IMU sensors and the haptic device, in the predefined tasks associated with different indices of difficulty (IDs). Error bars illustrate the data variances in a 95% confidence interval. It should be noted that the ranges of the raw data from multiple channels are largely different. Therefore, all measures are linearly transformed by min-max normalization for the ease of visualization and comparison, without the distortion of raw data. The entire range of values from minimum to maximum for each feature are mapped to the range from 0 to 1. The physiological response metrics, including EMG muscle activity, EEG cognitive states, galvanic skin conductance, and heart response, are shown in Fig 6A–6C, respectively; movement features in task space, path straight deviation and trajectory efficiency, are shown in Fig 6D; user limb motion kinematics of both forearm and upper arm, including angular velocity, linear acceleration, and RMS magnitude of jerk, are shown in Fig 6E and 6F, respectively. In addition, Pearson’s correlation coefficients, *r*, were reported to indicate the relationship between the features with respects to the predefined task difficulty index. Significance levels of correlation effects were determined via the *p*-value 0.05. Results of the correlation analysis for predictor variables with the significance values are reported in Table 2.

**Table 2:** Pearson correlation coefficient with *p*-value of candidate features extracted from multichannel measures.

Category	Var. of Interest	Corr. Coeff. with *p*	
		*r*	*p*
**Muscle Activity**	*PostD*_*rms*_	0.56	<0.001^†^
	*PectM*_*rms*_	0.61	<0.001^†^
	*PostD*_*MAV*_	0.50	<0.001^†^
	*PectM*_*MAV*_	0.54	<0.001^†^
**EEG-based Cognition**	*Engagement*	0.22	<0.001^†^
	*Workload*	0.23	<0.001^†^
	*Distraction*	0.17	<0.001^†^
	*SleepOnset*	0.15	0.002*
	*HeadMvL*	0.11	0.024**
**Skin Conductance**	*SC*_*vr*_	0.34	<0.001^†^
**Heart Rate**	*HR*	0.35	<0.001^†^
**Motion Kinematics**	*AngVel*_*FA*_	0.88	<0.001^†^
	*AngVel*_*UA*_	0.90	<0.001^†^
	*LinAcc*_*FA*_	0.70	<0.001^†^
	*LinAcc*_*UA*_	0.80	<0.001^†^
	*Jerk*_*FA*_	0.74	<0.001^†^
	*Jerk*_*UA*_	0.65	<0.001^†^
	*PathStrDev*	0.67	<0.001^†^
	*PathEff*	-0.83	<0.001^†^
**Task-specific Measure**	*MT*	0.93	<0.001^†^

Statistical significance of correlation is determined when *p* < 0.05.

*** *p* < 0.10

** *p* < 0.05

* *p* < 0.01

^†^ *p* < 0.001

Measured movement time (*MT*) using the haptic device was most significantly associated with the tasks difficulty indices, *p* < 0.001, with the highest correlation coefficient, *r* = 0.93, among all other metrics computed. More importantly, high correlation coefficients were found between the difficulty index and user motion kinematics, in both of end-effector task space and user space, ranging from 0.66 to 0.90, with significance value *p* < 0.001. Specifically, for movement measures in task space, in terms of the path straight deviation and path efficiency, significantly high correlations have been achieved between the task difficulty and the two, *r* = 0.67 and *r* = −0.83, respectively, *p* < 0.001. Furthermore, user physiological response demonstrates the similar trends with tasks in different difficulty levels, but slightly lower correlations, when compared to the motion characteristics. For EMG muscle activity, average correlation coefficients of RMS and MAV on both active muscles, posterior deltoid and pectoralis major, are 0.58 and 0.52, respectively, *p* < 0.001. Heart rate response was correlated with task difficulty in the moderate levels, *r* = 0.35, followed by the variance of skin conductance (*SC*_*vr*_), *r* = 0.31, *p* < 0.001. All EEG-based cognitive metrics derived from the BIOPAC software are significantly correlated with task difficulty levels, *p* < 0.05. Among these measures, the metrics *Engagement* and *Workload* show the moderate correlation with difficulty, with the correlation coefficients *r* = 0.22, and *r* = 0.23, respectively. Three cognitive features, *Distraction*, *SleepOnset*, and *HeadMvL*, however, reveal correlations less than 0.20, and thus excluded from the following regression modeling.

### Model evaluation

In this section, the overall accuracy of each of the four difficulty models is investigated. After data acquisition and pre-processing, a total of 420 observation sample instances were obtained in our database. According to the correlation-based feature selection and feature-level fusion, Table 3 shows the excluded and included predictor variables for regression modeling, as a result of the feature selection procedure. Aggregated accuracy results calculated from the 10-fold cross-validation were reported in Table 4, regarding the aforementioned deterministic criteria. Regression results of the individual modality (Model *III*: *kinm* and Model *IV*: *physio*) and their fusion (Model *II*: *fusion*) were reported, in comparison with the conventional Fitts’ law (Model *I*: *MT*).

**Table 3:** Overview of excluded and included predictor variables in the feature-level fusion for regression modeling.

Var. of Interest	Excluded	Included			
		Set *I*	Set *II*	Set *III*	Set *IV*
*Distraction*	•				
*SleepOnset*	•				
*HeadMvL*	•				
*PostD*_*rms*_			•		•
*PectM*_*rms*_			•		•
*PostD*_*MAV*_			•		•
*PectM*_*MAV*_			•		•
*Engagement*			•		•
*Workload*			•		•
*SC*_*vr*_			•		•
*HR*			•		•
*AngVel*_*FA*_			•	•	
*AngVel*_*UA*_			•	•	
*LinAcc*_*FA*_			•	•	
*LinAcc*_*UA*_			•	•	
*Jerk*_*FA*_			•	•	
*Jerk*_*UA*_			•	•	
*PathStrDev*			•	•	
*PathEff*			•	•	
*MT*		•			

Four sets of metrics were chosen for each of the four models generated.

**Table 4:** Summary table showing the aggregated 10-fold cross-validation results based on PLS-R regression.

MODEL	*R*^2^	Testing				
		RMSE	MAE	NRMSE	NMAE	MAPE%
***I***: *MT*	0.847	0.666	0.541	0.095	0.077	14.43
***II***: *fusion*	**0.927**	**0.462**	**0.361**	**0.067**	**0.052**	**9.49**
***III***: *kinm*	0.921	0.481	0.380	0.069	0.054	9.97
***IV***: *physio*	0.516	1.184	0.963	0.169	0.138	27.47

Models were extracted using input predictor variables with different feature sets, which are defined in Table 3. Bold denotes the highest average accuracy regarding each statistic measure.

Overall, the *fusion* model, combining both physiological response and kinematic motion metrics, exhibited the highest accuracy in predicting the task difficulty. The coefficient of determination (*R*^2^) of *fusion* model is 0.927, with the best accuracy with respect to RMSE, MAE, and MAPE. In addition, *fusion* model, and *kinm* model, do not show significant differences on the ID prediction accuracy. This indicates the reduced subsets of predictors are able to predict task difficulty with the goodness-of-fit, though less explanatory predictors are included. The *kinm* model, compared with the other individual modalities, was able to show significantly increased accuracy, followed by *MT* and *physio*. In contrast, significant differences on detection accuracy between the movement time and other modalities (except for *physio*) have been found, where the improvement of higher accuracy was achieved with up to 30.63% and 27.78% in terms of *RMSE*. The comparisons of normalized accuracy measures, NRMSE and NMAE, in Table 4 could also confirm the above analysis of prediction accuracy.

## Discussion

As an important step in our analysis, we examined the relationship between the movement time and the difficulty index. It was found that the increase of movement time using the haptic device is essentially in proportion with the predefined difficulty index. This result shows that Fitts’ Law would be preserved in the control of the haptic device, consistent with prior studies showing the speed-accuracy trade-off in various robotic interfaces. Indeed, decreases of movement time have been reported to be associated with improved technical skills, control effectiveness [66] and subjectively perceived difficulty [67]. However, it should be emphasized that measure of movement time is insufficient to explain various aspects of motor difficulty, as shown in Table 4.

Consistent with our hypothesis, confirmation of the task difficulty differences was observed in the sensorimotor response of human users, from the aspects of physiological response and motion kinematics. Notably, the kinematic motion profiles, obtained from the movement in the user and task workspaces, provide the best predicitors of task difficulty. Specifically, for user limb motion, the produced movement amplitudes and smoothness show the direct one-to-one mappings onto the difficulty index. Tasks with higher difficulty are associated with increased angular velocity and linear acceleration. This increase in task difficulty is also reflected in the increase in the magnitudes of movement jerk. This observation indicates a significant challenge in maintaining smoothness of limb motion with higher task difficulty. For the motor performance measures in the task space, the correlation analysis shows a significant reduction of the trajectory effectiveness and decrease of the straightness of the entire end-effector paths, for the increased task difficulty. Gradually changing difficulty index would likely evoke adjustments of user movement patterns, and affect the overall user manipulability to control the device.

Similarly, user physiological response also demonstrates observable associations with task difficulty indices. It is clear that higher task difficulty levels significantly correlate with increased underlying muscular activity, user engagement, cognitive workload and their respective variances. One explanation for the results is that in order to reach far-away targets, participants have to engage more mentally in path planning, and allocate greater cognitive efforts and attention while maintaining performance accuracy. Here it should be noted, however, the measures of physiological response were generally less correlated with the difficulty index, in comparison with motion kinematics. An explantation for this is that varying the target distances has a direct and distinct impact on the kinematic motion behavior and motor performance; on the contrary, user physiological response may not be sensitive to the changes of difficulty index. This might be due to the nature of participant’s physiological activity, and relatively low signal resolution. Nevertheless, concerning the effects of IDs on the human cognition and the significant associations, it was confirmed that task difficulty index could serve as an objective indictor in revealing the user workload and dynamic fluctuations in cognition.

In addition to the above statistical analysis, models are constructed by PLS-R regression to explore the usefulness of using predictor variables in optimizing the identification of task difficulty. However, by comparing the results in Table 4, it is clear that multimodal fusion, combining user physiological response and motion characteristics, has the synergetic effect in improving the accuracy of prediction, and ultimately enhancing the value of information for various levels of task difficulty. User physiological response, in conjunction with kinematic motion analysis, was able to explain the motor difficulty of tasks and its variances more accurately. Of course, these improvements involve the increased cost of multiple sensors and higher computational load. The trade-off between predictive accuracy and model complexity is an important consideration for the designers of human-in-the-loop control systems.

A potential limitation of this study is the accuracy and robustness using partial least square regression in modeling the highly complex, and nonlinear relationships between the response and input predictor variables. Since several parameters demonstrated nonlinear or exponential relationships with the difficulty index, an improvement in predictive performance is expected by the adoption of advanced machine learning methods, such as neural networks. Moreover, further improvement could be made by improving the user motor capture system and processing methods. Various data processing techniques in both time and frequency domain, such as Power Spectral Density analysis (PSD), could be considered for better detection of user physiological feedback, and how the subjects coordinate their movements during human-robot interactions [16, 57, 68]. A larger group of participants would also better define these results.

Finally, it must be emphasized that the basic target-reaching tasks in this study could likely be largely different from the context of realistic complex tasks, in which human typically interact with robots to perform an unstructured manipulation. However, segments of user behaviors demonstrated similarity to the representative target-reaching motions [69, 70]. Additionally, the size of target, as one of control parameters, may affect user motion and physiological response in a real, physical system. Therefore, additional consideration is necessary when applying our model in broader robot-assisted cases with various kinds of manipulation. The target size will also be an important consideration for our future work. Regardless, results indicate a distinct advantage of using the multivariate data-driven approach to assess difficulty. Also, the features that have been used in this study are independent of task types, and thus have the potential to be applied more globally.

## Conclusion

Difficulty during human-in-the-loop control interactions is hard to define and measure objectively. In this paper, we present and evaluate a model to estimate task difficulty by leveraging Fitts’ Law. Findings of statistical results during typical reaching tasks confirm the correlations between user sensorimotor response metrics and task difficulty, *p* < 0.001. Motion kinematic metrics had the best predication of task difficulty, *R*^2^ = 0.921; a fusion of physiological metrics and motion kinematics are believed to provide richer source of information for identification of difficulty, *R*^2^ = 0.927, with 30.63% improvement of predictive accuracy in comparison with the movement time model.

Overall, the task difficulty models presented in this paper, and the method used to develop them, provide useful insights into human response during human-in-the-loop control tasks. As our proposed models are independent of the task, they could be useful for the evaluation of more complex control tasks, such as teleoperated or shared control of robotic systems.
